# Validation of the IPSET score for thrombosis in patients with prefibrotic myelofibrosis

**DOI:** 10.1038/s41408-020-0289-2

**Published:** 2020-02-25

**Authors:** Paola Guglielmelli, Alessandra Carobbio, Elisa Rumi, Valerio De Stefano, Lara Mannelli, Francesco Mannelli, Giada Rotunno, Giacomo Coltro, Silvia Betti, Chiara Cavalloni, Maria Chiara Finazzi, Juergen Thiele, Mario Cazzola, Alessandro Maria Vannucchi, Tiziano Barbui

**Affiliations:** 1 Center Research and Innovation of Myeloproliferative Neoplasms (CRIMM), Department of Experimental and Clinical Medicine, Azienda Ospedaliera Universitaria Careggi, University of Florence, Florence, Italy; 2 FROM Research Foundation, Papa Giovanni XXIIII Hospital, Bergamo, Italy; 3 Division of Hematology, Fondazione IRCCS Policlinico San Matteo, and Department of Molecular Medicine, University of Pavia, Pavia, Italy; 4 Institute of Hematology, Catholic University, Fondazione Policlinico A. Gemelli IRCCS, Rome, Italy; 5 UOC Ematologia, ASST Papa Giovanni XXIII, Bergamo, Italy; 6 Institute of Pathology, University of Cologne, Cologne, Germany

**Keywords:** Translational research, Myeloproliferative disease

## Abstract

Pre-fibrotic myelofibrosis (pre-PMF) and essential thrombocythemia (ET) are characterized by similarly increased rate of thrombotic events, but no study specifically analyzed risk factors for thrombosis in pre-PMF. In a multicenter cohort of 382 pre-PMF patients collected in this study, the rate of arterial and venous thrombosis after diagnosis was 1.0 and 0.95% patients/year. Factors significantly associated with arterial thrombosis were age, leukocytosis, generic cardiovascular risk factors, *JAK2*V617F and high molecular risk mutations, while only history of previous thrombosis, particularly prior venous thrombosis, was predictive of venous events. The risk of total thromboses was accurately predicted by the the international prognostic score for thrombosis in essential thrombocythemia (IPSET) score, originally developed for ET, and corresponded to 0.67, 2.05, and 2.95% patients/year in the low-, intermediate-, and high-risk categories. IPSET was superior to both the conventional 2-tiered score and the revised IPSET in this cohort of pre-PMF patients. We conclude that IPSET score can be conveniently used for thrombosis risk stratification in patients with pre-PMF and might represent the basis for individualized management aimed at reducing the increased risk of major cardiovascular events. Further refinement of the IPSET score in pre-PMF might be pursued by additional, prospective studies evaluating the inclusion of leukocytosis and/or adverse mutational profile as novel variables.

## Introduction

The revised 2016 World Health Organization (WHO) classification of myeloproliferative neoplasms recognized prefibrotic myelofibrosis (pre-PMF) as an entity distinct from essential thrombocythemia (ET)^1–3^. While the initial presentation of pre-PMF is often isolated thrombocytosis, thereby mimicking ET, the course of pre-PMF may be highly symptomatic, and outcome is worse than ET^4,5^. Patients with pre-PMF have higher leukocyte and platelet counts, lower hemoglobin, higher lactate dehydrogenase levels, and more frequently splenomegaly compared with patients with ET^4^. In the largest series available, including 661 patients with pre-PMF, the median overall survival (OS) of pre-PMF was 14.7 years, significantly shorter than 30.2 years in a parallel cohort of 421 unselected patients with ET, accounting for a hazard ratio (HR) of 2.7 (95% confidence interval (CI), 1.9–3.7)^4^. Conversely, ET and pre-PMF have comparable risk of cardiovascular events. In one study that considered 180 patients with pre-PMF, the cumulative risk of thrombotic complications was 25.4% compared with 21.5% in 891 ET patients, accounting for a rate of overall major thrombosis of 1.9 and 1.7% patients/year, respectivey^6^. In another study on 109 pre-PMF patients, the 10-year cumulative incidence of thrombosis was 18.5% (95%CI, 10.7–27.8) compared with 18% (95%CI, 0.4–6.0) in 269 patients with ET^7^.

Prediction of thrombosis risk in ET currently relies on two different models. The conventional two-tiered model includes age >60 years and history of thrombosis, and stratifies patients in a low- and high-risk category with rate of thrombotic events of 0.95 and 2.86% patients/year^8^. More recently, following elucidation of the association of driver mutations with thrombotic risk^9,10^, a new scoring system, the international prognostic score for thrombosis in essential thrombocythemia (IPSET), was developed; IPSET enlists age >60 years, thrombosis history, cardiovascular risk factors and *JAK2*V617F mutation as the variables that allowed to separate patients into a low, intermediate, and high-risk category, with respective risk of thrombosis of 1.03, 2.35, and 3.56% patients/years^8,11^. The IPSET score was validated in an external validation cohort of 329 patients^8^ as well as an independent series of 585 patients from the Mayo Clinic^12^; it was also validated in a population of ET patients from China^13^. IPSET was subsequently revised to identify a very low-risk category of patients, which includes younger patients without history of thrombosis and lacking the *JAK2*V617F mutation^12,14^. The IPSET-revised and/or IPSET is recommended by recent guidelines, including those from the European Leukemia Net and the National Comprehensive Cancer Network, as the most appropriate risk scoring system(s) to personalize treatment with antiplatelet agents and/or cytoreduction, or follow up only, in patients with ET^15,16^. On the opposite, no score has been specifically developed to assess thrombotic risk in pre-PMF, reflecting difficulties and variability in the management of this condition by hematologists^17^. Furthermore, it has not been addressed yet whether the available scores for ET may be informative in patients with pre-PMF as well; this question was the objective of current work.

## Materials and methods

### Study population

Clinical and hematologic information of 382 consecutive patients with a diagnosis of pre-PMF were collected from four Italian tertiary centers (Florence, Pavia, Bergamo, Rome). Patients’ eligibility criteria included revised diagnosis according to 2016 WHO criteria and known genotype for *JAK2*V617F, *MPL*W515L/K, Calreticulin (*CALR*) mutation^1^. The study was approved by each Institutional Review Board, and was conducted according to Helsinki declaration; written, informed consent was obtained from all living subjects. In a group of patients (*n* = 132), information regarding high molecular risk (HMR) mutations, including *ASXL1, EZH2, SRSF2, IDH1/2*, were available; there was no specific predefined criteria for HMR analysis, so these patients were randomly analyzed in the different centers’ cohorts. Histopathology, hematologic and clinical data were carefully reviewed in each case and diagnosis was confirmed based on the revised 2016 WHO criteria. Histopathology analysis was performed locally, blinded of patient’s history and clinical information except for sex and age. Clinical and hematologic information were coincident (±12 months) with the diagnostic biopsy.

### Mutation analysis

Analysis was performed on DNA from peripheral blood granulocytes collected at diagnosis or within 1 year, as concerned driver mutations, and up to 4 years after diagnosis (median, 19 months, range 0–46 months) for HMR mutations. *JAK2*V617F mutation was assessed by real-time quantitative PCR; for *MPL* mutations, high-resolution melting analysis and bidirectional Sanger sequencing were employed; *MPL*515x indicates any mutation at codon 515^9^. *CALR* mutations were identified by capillary electrophoresis and bidirectional sequencing, and classified as type 1/type 1-like or type 2/type 2-like^18,19^. Patients lacking mutations in the three driver genes were defined as “triple negative” (TN). A next generation sequencing approach with the PGM Ion Torrent platform was used to detect mutations across the entire coding region of *EZH2* and *ASXL1*, and mutation hotspots for *IDH1*, *IDH2*, and *SRSF2*. An HMR category was defined by presence of at least one of the above mutations, as reported previously^20,21^; information on other myeloid neoplasm associated mutations with potential prognostic impact, such as *U2AF1*^22^, were not available. In case of variants not previously reported, only those considered potentially damaging by Polyphen and SIFT algorithm were included in the database. Genotyping for driver mutations was performed locally, while analysis of HMR mutations was centralized (CRIMM, Florence).

### Statistical analysis

Thrombosis‐free survival was determined from diagnosis to the first thrombotic event. In case of thrombotic events before establishment of diagnosis of pre-PMF, only those occurred in the previous 3 years were considered. Cox-regression analysis was used for multivariable analysis. Patients were stratified according to the conventional two-tiered risk stratification system^23^, IPSET‐thrombosis^8^ and revised IPSET‐thrombosis^14^ model. Harrell’s concordance (C) statistic was calculated to measure the predictive accuracy of different models. A *P* < 0.05 was considered statistically significant.

## Results

A total of 382 patients were included; their clinical and hematologic characteristics at diagnosis are reported in Table 1. Median age was 57.6 years, 45% were above the age of 60; 51% were male. Generic cardiovascular risk factors were present in 45%: smoking (14%), hypertension (27%), diabetes mellitus (8%), and hypercholesterolemia (12%). A driver mutation was found in 92%: *JAK2*V617F in 65%, *MPL*W515L/K 4.5%, *CALR* type1 16.5%, and *CALR* Type2 6%; 31 patients (8%) were TN. HMR mutations were available in 132 patients (35%); of these, 28% were HMR positive, and 8% had >2 HMR mutations (Table 2). Overall, 239 patients (77%) received a cytoreductive treatment; hydroxyurea was the most commonly used (68%). Antiplatelet agent was used in 72% of the patients (acetylsalicylic acid 88%, clopidogrel 10%, ticlopidine 1%), while 57 patients (15%) received anticoagulant agents (vitamin K antagonists 75%, direct oral anticoagulants 12%, heparin 11%) (Supplementary Table 1). After a median follow-up of 6.9 years (range, 0.08–32.6), 105 patients (27%) died. Sixty patients (16%) experienced overt myelofibrotic progression and 29 (8%) transformed to acute leukemia, with incidence rate of 2.05%, 0.95% and 3.41% patients/year, respectively (Table 3). Major hemorrhages affected 3 and 7% of the patients before/at diagnosis and during follow-up, respectively.

**Table 1:** Clinical and hematologic characteristics at diagnosis of 382 study patients with prefibrotic PMF.

Variables	*N*	
Male, *n* (%)	382	195 (51%)
Age, years, median (range)	382	57.6 (15.6–91.9)
Age ≥60 years, *n* (%)	382	170 (45)
Previous thrombosis, *n* (%)	382	65 (17)
Arterial		35 (9)
Venous		31 (8)
Microvascular disturbances, *n* (%)	370	63 (17)
Major bleeding, *n* (%)	382	11 (3)
Blood values		
Hemoglobin, g/L, median (5th–95th percentiles)	376	135 (93–159)
Hematocrit, %, median (5th–95th percentiles)	345	41.1 (30.6–49.0)
Platelet count, ×10^9^/L, median (5th–95th percentiles)	380	700 (130–1481)
Leukocyte count, ×10^9^/L, median (5th–95th percentiles)	378	10.0 (5.0–24.1)
LDH > normal range; *n* (%)	300	220 (73)
Bone marrow fibrosis grade, *n* (%)	382	
0		161 (42)
1		221 (58)
Palpable splenomegaly, *n* (%)	382	180 (47)
Spleen size, cm from LCM, *n* (%)	382	
<5 cm		42 (11)
6–10 cm		42 (11)
11–15 cm		71 (19)
16–20 cm		44 (12)
>20 cm		23 (6)
CV risk factors (at least 1), *n* (%)	360	161 (45)
History of active/remote smoking		50 (14)
History of hypertension		97 (27)
History of diabetes mellitus		27 (8)
History of hypercholesterolemia		39 (12)
Thrombophilia, *n* (%)	153	
Inherited		19^a^ (12)
Acquired		30^a^ (19)
Negative		105 (69)
Abnormal cytogenetics, *n* (%)	286	42 (15)

^a^One patient had both inherited and acquired thrombophilia.

*LCM* left costal margin; *CV* cardiovascular.

**Table 2:** Mutation profile of study patients.

Variables	*N*	
Driver mutation		
*JAK2*V617F, *n* (%)	378	246 (65)
VAF	225	36.8 (0.3–100)
*CALR* type1, *n* (%)	288	63 (22)
VAF	44	50 (9–63.7)
*CALR* type2, *n* (%)	134	24 (18)
VAF	21	49 (7–94)
*MPL* W515x, *n* (%)	336	17 (5)
Triple negatives, *n* (%)	377	31 (8)
Non-driver mutations		
*ASXL1*, *n* (%)	133	28 (21)
*EZH2*, *n* (%)	132	5 (4)
*SRSF2*, *n* (%)	132	14 (11)
*IDH1/2*, *n* (%)	132	1 (1)
HMR, *n* (%)	132	37 (28)
HMR ≥ 2, *n* (%)	132	11 (8)

Data are reported as median (range).

*VAF* variant allele frequency, *HMR* high molecular risk, points to the presence of at least one mutation in any one of ASXL1, EZH2, SRSF2, IDH1/2. HMR ≥ 2 means the presence of two or more mutated genes among the above. Two or more mutations in the same gene are counted as one.

**Table 3:** Major clinical events after diagnosis and outcome in the study population (*n* = 382).

Events	
Major thrombosis, *n* (%)	56 (15)
Rate % pts/year, (95% CI)	1.99 (1.53–2.60)
Arterial, *n* (%)	30 (8)
Rate % pts/year, (95% CI)	1.00 (0.70–1.45)
Venous, *n* (%)	28 (7)
Rate % pts/year, (95% CI)	0.95 (0.66–1.38)
Major bleeding, *n* (%)	28 (7)
Rate % pts/year, (95% CI)	0.94 (0.65–1.36)
Overt MF, *n* (%)	60 (16)
Rate % pts/year, (95% CI)	2.05 (1.59–2.64)
AML, *n* (%)	29 (8)
Rate % pts/year, (95% CI)	0.95 (0.66–1.36)
Death, *n* (%)	105 (27)
Rate % pts/year, (95% CI)	3.41 (2.81–4.13)

Two patients had both arterial and venous events.

A major thrombotic event occurred before/at diagnosis in 65 patients (17%), including 35 arterial (9%), and 31 venous (8%) thromboses. During the follow-up, 56 patients (15%) developed a thrombotic event, with incidence rate of 1.99% patients/year (95% CI 1.53–2.60); 30 were arterial (8%) and 28 venous (7%) events, accounting for incidence rate of 1.00% (95% CI 0.70–1.45) and 0.95% (95% CI 0.66–1.38) patients/year, respectively (Table 4). The most frequent arterial thrombosis were acute myocardial infarction and stroke (23% each at diagnosis, 30% and 27%, respectively, during follow up). As regarded venous events, splanchnic vein thrombosis (SVT) represented as many as 68% of all venous events at diagnosis, and 29% during follow up, whereas deep venous thrombosis and pulmonary embolism accounted for 43% of all venous events during follow up (Table 4). In univariate analysis, factors significantly associated with arterial thrombosis were age greater than 65 years (HR 2.88, 95%CI 1.37–6.05; *P* = 0.005), leukocyte count >10 × 10^9^/L (HR 2.43, 95%CI 1.11–5.31; *P* = 0.026), presence of at least one CV risk factor (HR 2. 16, 95%CI 1.01–4.62; *P* = 0.047), *JAK2*V617F mutation (HR 3.35, 95%CI 1.15–9.74; *P* = 0.027), HMR status (HR 13.1, 95% CI 1.34–127; *P* = 0.027), and *EZH2* mutation (HR 10.1, 1.03–99; *P* = 0.027). Conversely, only history of previous thrombosis retained significance for venous thrombosis (HR 3.06, 95% CI 1.41–6.64; *P* = 0.005), particularly in case of a previous venous event (HR 5.53, 95% CI 2.32–12.20; *P* < 0.0001). Male sex showed a trend (*P* = 0.079) toward protection for venous events (HR = 0.49; 95%CI, 0.22–1.09) (Table 5).

**Table 4:** Type of thrombotic events occurring before/at diagnosis and during follow-up.

	Before/At diagnosis	During follow-up
Arterial thrombosis, *N*	35	30
AMI	8 (23%)	9 (30%)
Stroke	8 (23%)	8 (27%)
TIA	7 (20%)	5 (17%)
PAT	7 (20%)	4 (13%)
Abdominal	4 (11%)	1 (3%)
Other^a^	1 (3%)	2 (7%)
Venous thrombosis, *N*	31	28
DVT/PE	5 (16%)	12 (43%)
Budd-Chiari	3 (10%)	2 (7%)
SVT	21 (68%)	8 (29%)
Other^b^	2 (6%)	−

*AMI* acute myocardial infarction, *TIA* documented transient ischemic attack, *PAT* peripheral arterial thrombosis, *DVT/PE* deep venous thrombosis/pulmonary embolism, *SVT* splanchnic vein thrombosis, including porto-mesentheric, splenic, splanchnic venous thrombosis.

^a^Lung, retinal.

^b^Cerebral, retinal thrombosis.

**Table 5:** Predictors* of arterial, venous and total thrombosis in univariate analysis.

	Arterial thrombosis (*n* = 30)		Venous thrombosis (*n* = 28)	
Variables	HR (95% CI)	*P* value	HR (95% CI)	*P* value
Male sex			0.49 (0.22–1.09)	0.079
Age, years	1.04 (1.01–1.07)	0.004		
≥60 years	2.01 (0.96–4.21)	0.062		
≥65 years	2.88 (1.37–6.05)	0.005		
Previous thrombosis			3.06 (1.41–6.64)	0.005
Arterial				
Venous			5.53 (2.32–12.2)	<0.0001
Leukocyte count ≥10×10^9^/L	2.43 (1.11–5.31)	0.026		
CV risk factors^a^ (at least 1)	2.16 (1.01–4.62)	0.047		
Driver mutations				
*JAK2*V617F	3.35 (1.15–9.74)	0.027		
Nondriver mutations				
EZH2	10.1 (1.03–99)	0.047		
HMR	13.1 (1.34–127)	0.027		

^*^Variables with a *P* < 0.10 in univariate analysis.

^a^History of smoking, hypertension, diabetes mellitus, hypercholesterolemia.

**Fig. 1:**
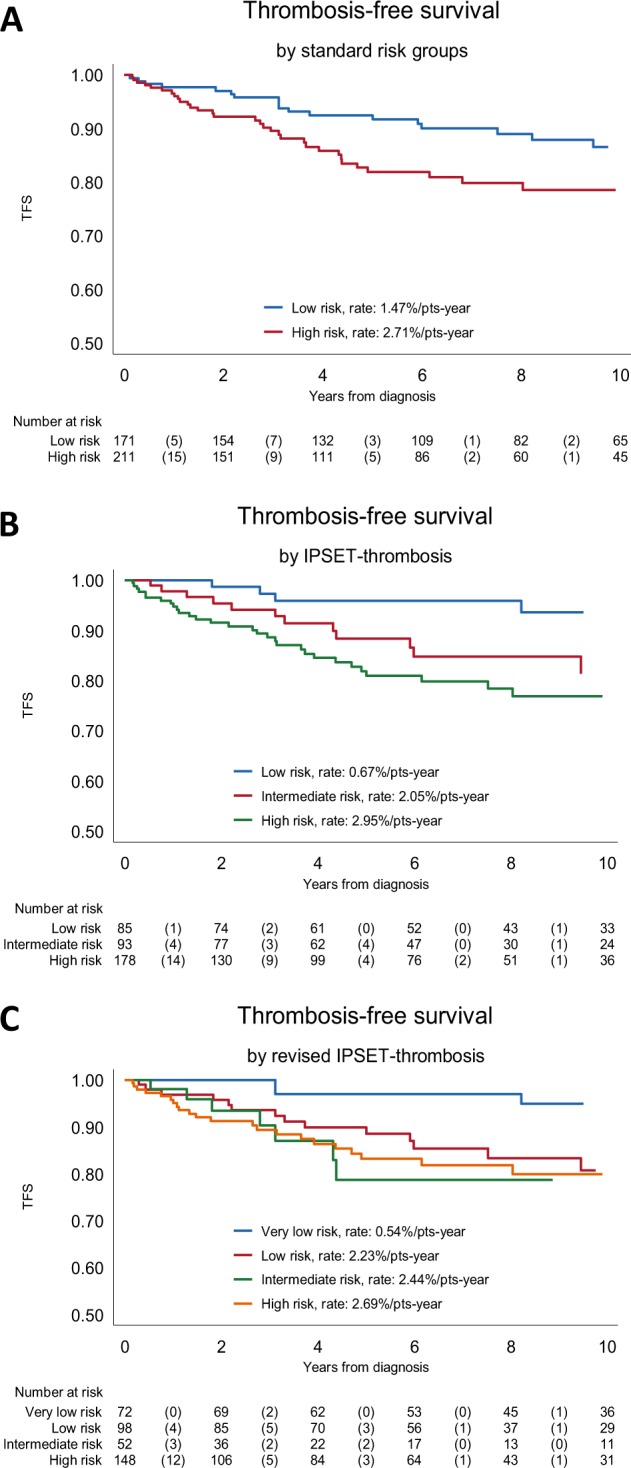
Thrombosis-free Survival Analysis. The Kaplan–Meyer analysis of thrombosis free survival (TFS), considering all thrombotic events after diagnosis in the 382 patients included in the study, are shown in **a**, according to the conventional 2-tiered risk score (**b**) for the IPSET score, and **c**, for the revised IPSET score.

To evaluate the performance of available risk scores for predicting thrombosis in pre-PMF, patients were first stratified according to the conventional two-tiered risk score. A statistically significant difference between low- and high-risk category was observed, pointing to an incidence rate of 1.47% and 2.71% patients/year, respectively (*P* = 0.041) (Fig. 1, panel A); the HR for the high-risk versus low-risk category was 1.74 (95% CI 1.02–2.99; *P* = 0.044). Then, patients were stratified according to IPSET and revised IPSET model, resulting in effective discrimination of different categories. The rate of thrombosis in IPSET low-, intermediate-, and high-risk category was 0.67, 2.05 and 2.95% patients/year (*P* = 0.006); the HR was 2.81 and 4.14 for the intermediate- and high-risk versus reference low-risk category (Table 6). In the revised-IPSET very low-, low-, intermediate-, and high-risk category, the rate of thrombosis was 0.54%, 2.23%, 2.44% and 2.69% patients/year, respectively (*P* = 0.023), with HR of 3.90, 4.14, and 4.63 versus reference category. The corresponding thrombosis-free survival curves are shown in Fig. 1, panel B, C.

**Table 6:** Performance of prognostic risk stratifications models for thrombosis.

Prognostic model	*N* (%)		Total thrombosis (*n* = 56)	
		*N* thrombosis	HR (95% CI), *P* value	C-statistic
Standard risk groups^a^	382			0.58
Low risk	171 (45)	22	1 (ref)	
High risk	211 (55)	34	1.74 (1.02–2.99), 0.044	
IPSET-thrombosis risk groups^b^	356			0.63
Low risk	85 (24)	5	1 (ref)	
Intermediate risk	93 (26)	13	2.81 (1.00–7.91), 0.050	
High risk	178 (50)	32	4.14 (1.61–10.7), 0.003	
IPSET-thrombosis revised risk groups^c^	370			0.62
Very low risk	72 (19)	4	1 (ref)	
Low risk	98 (27)	17	3.90 (1.31–11.6), 0.014	
Intermediate risk	52 (14)	7	4.14 (1.21–14.2), 0.024	
High risk	148 (40)	24	4.63 (1.60–13.4), 0.005	

N(%) indicates the number of patients for whom all required data were available, and the % over total (*n* = 382).

^a^“low risk” (age ≤ 60 years and no thrombosis history); “high risk” (age ≥ 60 years and/or thrombosis history).

^b^“low risk” (tot score 0–1); “intermediate risk” (tot score 2); “high risk” (tot score ≥ 3). Age ≥60 years and at least 1 CV risk factor (1 score); thrombosis history and JAK2 mutation (2 scores).

^c^“very low risk” (no thrombosis history, age ≤60 years and JAK2-unmutated); “low risk” (no thrombosis history, age ≤60 years and JAK2-mutated); intermediate risk’ (no thrombosis history, age ≥60 years and JAK2-unmutated) and high risk (thrombosis history or age ≥60 years with JAK2 mutation).

**Fig. 2:**
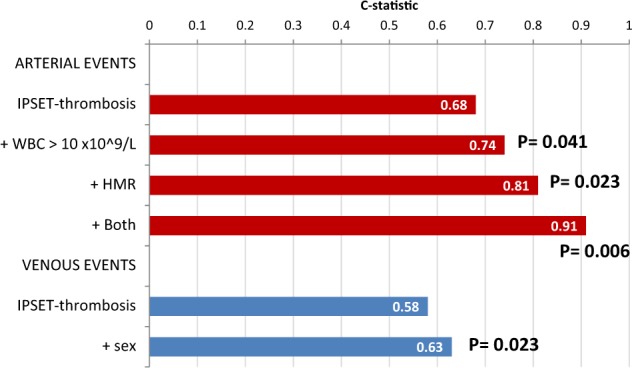
C-statistic Analysis. Incremental value of C-statistic by new risk factors added to the IPSET prognostic risk scoring model for arterial (leukocytosis, high mutation risk (HMR) status, and both) and venous (male sex) events. *P* values quoted are calculated testing the difference on coefficients of the Cox-regression models.

The incremental value of incorporating new risk factors that were identified in this study, and are not included in the original IPSET model, was also preliminary evaluated. When WBC > 10 × 10^9^/L (this threshold was preliminary defined by ROC analysis as the one having best discrimination power) and HMR status were incorporated into the model for arterial events, the C statistic increased significantly from the baseline value of 0.68–0.74 after the addition of leukocyte count >10 × 10^9^/L (*P* = 0.041), to 0.81 (*P* = 0.023) for HMR status and 0.91 (*P* = 0.006) when the HMR status plus leukocytosis was added to IPSET. Conversely, inclusion of male sex increased the *C* value of IPSET from 0.58 to 0.63 (*P* = 0.023) for venous thrombosis (Fig. 2).

## Discussion

Patients with prefibrotic myelofibrosis are at about twofold increased risk of cardiovascular events, including major thrombosis and hemorrhage, compared with the reference age-matched population, a figure that is substantially similar to patients with ET. Since in ET thrombosis represents the main cause of mortality, scores have been developed over time to predict the individual risk of thrombosis and facilitate the adoption of the most appropriate therapeutic measures. Patients with ET are conventionally stratified based upon older age and previous thrombosis, with an intermediate category including those with cardiovascular risk factors only. A new score was developed recently, the IPSET score, and a revised version (IPSET-revised), which takes advantage of the powerful prognostic value of the *JAK2*V617F mutation in addition to the classical risk variables^8,14^. On the other hand, there is little information regarding the variables that associate with thrombosis risk in patients with pre-PMF, translating in uncertainties regarding stratification and management of these patients^17,24^.

With the aim to approach this issue, we collected a multicenter cohort of 382, consecutive, patients with a diagnosis of pre-PMF, carefully revised according to the 2016 WHO criteria, and we analyzed major thrombotic events at diagnosis and during the follow-up by employing the conventional risk score and the IPSET and IPSET-revised score. Main results of this analysis indicate that the IPSET score, and to a somewhat lesser degree (as indicated by the HR values of the individual risk categories; Table 6) the IPSET-revised, effectively discriminates patients with pre-PMF in a low, intermediate (HR, 2.81 versus low-risk) and high-risk (HR 4.14) category. The rate of thrombosis in these categories is substantially similar to those reported in patients with ET^8,12,13^, and are about two- and three-fold higher than expected in normal population.

Based on univariate analysis indicating that, in addition to the variables enlisted in the IPSET score, also leukocytosis and an HMR status for arterial events, and male sex for venous events, correlated with thrombosis risk, we evaluated their impact in the settings of the IPSET score itself, showing that, at variable extent, all increased the predictive power of the model. In particular, we noticed with interest the novel finding that the HMR status, known to be highly informative in patients with overt MF as concerns OS and transformation to leukemia^20,21,25,26^, represented a powerful risk factor for thrombosis in the settings of pre-PMF, and increased significantly the predictive power of the IPSET model from 0.68 to 0.91, as assessed by C-statistics. Among the genes constituting the HMR category, *EZH2* emerged as the most statistically significant one in univariate analysis, but due to the low number of cases it was not assessed in multivariate analysis. However, we underline that the HR for thrombosis of HMR mutations was wide, likely owing to the low number of patients with the mutation(s) and concurrent thrombotic events; these findings clearly need to be substantiated in larger studies, with the possibility that a revised IPSET specifically developed for pre-PMF patients might benefit from addition of one or both variables.

We acknowledge that there are intrinsic limitations to this study, which include the lack of an independent validation cohort, a central review of histopathological specimens, yet evaluated by expert local hemopathologists strictly adhering to 2016 WHO criteria, and the restriction of analysis of HMR mutations to a subset only of the entire population of study; both could be the objective of future studies. However, as regards HMR mutations, we are quite confident about the lack of any population selection bias owing that the overall frequency of individual mutations was overall comparable to what reported in another series of 278 pre-PMF patients^4^. We also acknowledge that more extensive mutation analysis, using gene panels larger than the one originally designed for assessing HMR mutations, could permit to identify novel somatic variables associated with thrombotic risk in pre-PMF patients, for example *TET2* mutations as shown in subjects with age-related clonal hematopoiesis^27^. Finally, we cannot exclude at this time that the high proportion of splancnic thrombosis among the venous events recorded at diagnosis represents a bias of referral due to the tertiary nature of the involved centers, an observation that needs however to be revisited. This high rate of occurrence might also explain the finding of protective effects of male sex for venous thrombosis, owing that SVT occurred in females more than 90% of cases.

In conclusion, we report that patients with pre-PMF can be accurately stratified in different risk categories for cardiovascular events using the IPSET score as developed for ET; possible refinements might derived from the inclusion of leukocytosis^28^ and/or additional molecular variables^29^, such as HMR mutations, and possibly other mutations. Also, whether the same management approach to minimize the risk of first or recurrent thrombosis that are used for ET patients, based on the IPSET stratification, results similarly effective in patients with pre-PMF remains a research question for future studies^17,30^.

## Supplementary information

Supplemental Material
