# Supplementary material for: Validation of the IPSET score for thrombosis in patients with prefibrotic myelofibrosis
Source: Blood Cancer J. 2020 Feb 25;10(2):21. doi: 10.1038/s41408-020-0289-2 (PMC7042364; doi:10.1038/s41408-020-0289-2)
Supplement: Supplementary file 1 — Supplemental Material [file 41408_2020_289_MOESM1_ESM.docx]

**Supplemental material to:**

**Validation of the international prognostic score for thrombosis in essential thrombocythemia (IPSET) in patients with prefibrotic myelofibrosis.**

**Author:** Paola Guglielmelli et al.

**Supplemental Table 1. Treatment.** Treatment details of 382 consecutive patients with a diagnosis of pre-PMF

| **Treatments** | **N** |  |
| --- | --- | --- |
| Chemotherapy-free | 381 | (23%) |
| Chemotherapy  Hydroxyurea  Interferon  Anagrelide  Ruxolitinib  Pipobroman  Busulfan  Everolimus  Thalidomide  *Combinations* | 381 | 293 (77)  199 (68)  11 (4)  7 (2)  6 (2)  3 (1)  2 (0.5)  2 (0.5)  1 (0)  *61 (21)* |
| Antiplatelets, n (%)  ASA  CLOPIDOGREL  TICLOPIDINA  *Combinations* | 377 | 273 (72)  237 (88)  27 (10)  4 (1)  2 (1) |
| Anticoagulants, n (%)  VKA  DOACs  HEPARIN  *Combinations* | 378 | 57 (15)  43 (75)  7 (12)  6 (11)  1 (2) |
